# Pain Awareness Month — September 2018

**DOI:** 10.15585/mmwr.mm6736a1

**Published:** 2018-09-14

September is Pain Awareness Month, when organizations work to raise awareness of how pain affects persons, families, communities, and the nation and to support national action to address pain. A 2011 Institute of Medicine report (https://www.ncbi.nlm.nih.gov/pubmed/22553896) has prompted strategic planning efforts, such as the 2016 National Pain Strategy (https://iprcc.nih.gov/sites/default/files/HHSNational_Pain_Strategy_508C.pdf) and the 2017 Federal Pain Research Strategy (https://iprcc.nih.gov/Federal-Pain-Research-Strategy/Overview), and efforts for their implementation.

A report on chronic pain in this issue (*1*) estimates that chronic pain affects approximately 50 million U.S. adults, and high-impact chronic pain (i.e., interfering with work or life most days or every day) affects approximately 20 million U.S. adults. Findings in this report will help guide federal efforts to address high-impact chronic pain, such as *Healthy People 2020* objectives (https://www.healthypeople.gov/2020/topics-objectives) and the CDC Guideline for Prescribing Opioids for Chronic Pain (https://www.cdc.gov/mmwr/volumes/65/rr/rr6501e1.htm). Better public education regarding expectations, beliefs, and understanding about pain are all important. Additional measures include professional education and training for better, comprehensive, and integrated pain management.

Better pain management is also a major element in addressing the current opioid crisis. Persons living with pain need safer and more effective alternatives for pain management. Additional information is available at https://www.hhs.gov/opioids/about-the-epidemic/hhs-response/better-pain-management/index.html.
